# Mitochondria Homeostasis and Oxidant/Antioxidant Balance in Skeletal Muscle—Do Myokines Play a Role?

**DOI:** 10.3390/antiox10020179

**Published:** 2021-01-27

**Authors:** Brian Pak Shing Pang, Wing Suen Chan, Chi Bun Chan

**Affiliations:** School of Biological Sciences, The University of Hong Kong, Hong Kong; ppsb333@hku.hk (B.P.S.P.); suencws@hku.hk (W.S.C.)

**Keywords:** aging, exercise, mitochondria, myokine, ROS

## Abstract

Mitochondria are the cellular powerhouses that generate adenosine triphosphate (ATP) to substantiate various biochemical activities. Instead of being a static intracellular structure, they are dynamic organelles that perform constant structural and functional remodeling in response to different metabolic stresses. In situations that require a high ATP supply, new mitochondria are assembled (mitochondrial biogenesis) or formed by fusing the existing mitochondria (mitochondrial fusion) to maximize the oxidative capacity. On the other hand, nutrient overload may produce detrimental metabolites such as reactive oxidative species (ROS) that wreck the organelle, leading to the split of damaged mitochondria (mitofission) for clearance (mitophagy). These vital processes are tightly regulated by a sophisticated quality control system involving energy sensing, intracellular membrane interaction, autophagy, and proteasomal degradation to optimize the number of healthy mitochondria. The effective mitochondrial surveillance is particularly important to skeletal muscle fitness because of its large tissue mass as well as its high metabolic activities for supporting the intensive myofiber contractility. Indeed, the failure of the mitochondrial quality control system in skeletal muscle is associated with diseases such as insulin resistance, aging, and muscle wasting. While the mitochondrial dynamics in cells are believed to be intrinsically controlled by the energy content and nutrient availability, other upstream regulators such as hormonal signals from distal organs or factors generated by the muscle itself may also play a critical role. It is now clear that skeletal muscle actively participates in systemic energy homeostasis via producing hundreds of myokines. Acting either as autocrine/paracrine or circulating hormones to crosstalk with other organs, these secretory myokines regulate a large number of physiological activities including insulin sensitivity, fuel utilization, cell differentiation, and appetite behavior. In this article, we will review the mechanism of myokines in mitochondrial quality control and ROS balance, and discuss their translational potential.

## 1. Introduction

Skeletal muscle is the largest organ in the human body in terms of tissue mass. In addition to supporting locomotion, it is a metabolic reservoir of amino acids and postprandial glucose [1,2]. A finding in the 60s proposed that skeletal muscle might actively participate in systemic metabolic regulation possibly by releasing humoral hypoglycemic factors [3]. This idea of “myokine” was finally established in 2003 after interleukin 6 (IL-6) secretion from skeletal muscle during exercise was confirmed [4]. By definition, myokines are cytokines or peptides that are produced, expressed, and released by muscle fibers to induce functional changes in tissues via cross-talking with other organs or acting locally on skeletal muscle. The functions of myokines are multitudinous that include metabolic regulation in liver and pancreas, remodeling of adipose tissues, improvement of cognitive function and neurogenesis in the central nervous system (CNS), bone mass maintenance, angiogenesis regulation, and immunomodulation [5,6]. Using mass spectrometry-based proteomics and in silico genomic analysis, it is estimated that ~650–1000 proteins/factors can be found in the skeletal muscle [7]. The number of potential myokines can be as much as 1000 if the presence of secretory signal peptide is set as a selection criterion. Nevertheless, less than 10% of the identified myokines have been functionally and mechanistically characterized, which leaves a huge knowledge gap in the area of endocrinology and metabolism research.

Mitochondria are the central organelles of skeletal muscle metabolism. A large amount of evidence has shown that maintaining the normal function of mitochondria is essential for the general health of an organism, and dysfunction of mitochondrial activity is tightly associated with diseases such as muscle wasting and metabolic syndromes [8,9]. Due to the improvement in gene manipulation, biochemical detection, metabolomics, proteomics, and genomic analyses, our understanding of the mitochondria activity in various physiological and disease conditions has been greatly advanced. It is now clear that the activity of mitochondria could be regulated by numerous intracellular and external factors such as nutrient availability and exercise status. However, the relationship between myokines and mitochondrial activity is still being established.

In addition to adenosine triphosphate (ATP) generation, mitochondria also produce reactive oxygen species (ROS) as a side product. ROS is a group of oxygen-derived molecules and free radicals that may bring about oxidative damage to muscle fibers via apoptosis, autophagy, inflammation, and atrophy. Nevertheless, the presence of ROS is essential in maintaining muscle functions such as myocyte repair, mitochondria biogenesis, muscle survival, and muscle maturation [10]. A highly regulated antioxidation system is thus indispensable to balance the ROS content in muscles. Although numerous modulatory factors on oxidative stress regulation have been identified, less is known about the relationship between myokine and ROS/antioxidation in muscle. In this review, we will discuss the functional and mechanistic actions of myokines in mitochondrial recycling and reactive oxidative species homeostasis.

## 2. Myokines and Mitochondrial Dynamics

Due to the high activity of oxidative phosphorylation, mitochondria are faced with oxidative damages regularly. This oxidative menace is increased when the tissues are required to handle a large amount of nutrient input during obesity or stressed by the demand for extra ATP during physical exercise. Consequently, the mitochondrial pool has to undergo regular recycling to replace the damaged organelle with new and functional mitochondria, which is a critical event to maintain the functionality of cells.

The life cycle of mitochondria includes mitochondrial biogenesis, mitofusion, mitofission, and mitophagy. The majority of new mitochondria are synthesized through the nucleus-initiated “mitochondrial biogenesis” or “retrograde signaling” from mitochondria [11]. In situations where a prolonged supply of additional ATP is required (e.g., exercise and cold adaptation), the transcriptional co-activator peroxisome proliferator-activated receptor-γ coactivator 1α (PGC-1α) plays the master role of nucleus-initiated mitochondrial biogenesis [12]. Activated by the energy-sensing kinase AMP-activated protein kinase (AMPK) [13,14] and cooperating with another transcription factor nuclear respiratory factor 1 (NRF1), PGC-1α induces the expression of genes such as mitochondrial transcription factor A (mtTFA) for promoting the synthesis of new mitochondrial proteins [15]. The activity of PGC-1α is also regulated by another energy sensor sirtuin 1 (SIRT1) in response to low glucose availability. This NAD^+^-dependent deacetylase activates PGC-1α by deacetylation, leading to elevated expression of mitochondrial proteins and β-oxidation-related genes [16]. Apart from the cellular energy status, mitochondria biosynthesis can also be initiated by the damaged mitochondria [17]. The disrupted membrane potential of deformed mitochondria, which is associated with Ryr-1-dependent increase of cytosolic [Ca^2+^], activates calcineurin-dependent nuclear factor of activated T-cells (NFATc), myocyte enhancer factor 2 (MEF2), and c-Jun N-terminal kinase (JNK)-dependent activating transcription factor 2 (ATF2) pathways to trigger new mitochondria synthesis [18].

Mitochondrial fusion improves the capacity of oxidative phosphorylation and allows redistribution of mitochondrial DNA (mtDNA) between the healthy and damaged mitochondria [19]. It is also an important process to protect the elongated mitochondria from non-selective degradation by autophagy during energy scarcity [20]. The regulatory mechanism driving mitofusion is highly conserved among species. In mammals, the dynamin-like GTPases mitofusins (MFN1 and MFN2) and optic atrophy protein 1 (OPA1) are the major mediators of mitochondrial fusion. Loss-of-function studies showed that trans-interaction of MFNs brings together mitochondrial outer members to trigger the fusion of two individual mitochondria [21]. Similarly, OPA1 forms oligomers in the inner mitochondrial membrane to promote membrane curvature and lipid mixing during membrane fusion [22]. Because mitochondrial fusion reduces the mobility of mitochondria, permanent fusions are rare events and less than 10% of mitochondria undergo complete fusion. Instead, mitochondria often adopt transient fusions so that two mitochondria are temporarily merged to exchange intra-matrix materials, which are then separated to their original form [23].

Despite that mitochondria fusion is remedial to stressed cells, there is a limit to the stress level [24]. If the threshold is reached, the mitochondria will undergo “mitofission” in which the organelle split asymmetrically into a “healthy” mother mitochondria and a “damaged” daughter mitochondria with disrupted membrane potential [25,26]. Mitofission is mainly mediated by dynamin-related protein 1 (DRP1), a GTPase of the dynamin family that normally resides in the cytoplasm [27]. When recruited by mitochondrial membrane receptors such as mitochondrial fission protein 1 (Fis1) or mitochondrial fission factor (Mff), DRP1 forms spiral oligomers that constrict the mitochondrial membrane upon GTP hydrolysis [28,29]. The mitofission-stimulatory role of DRP1 also depends on its phosphorylation status. During starvation, protein kinase A (PKA) phosphorylates DRP1 at Ser 637 to suppresses its GTPase activity, which keeps the mitochondria in the fused state [30]. When mitofission is prohibited permanently in muscle-specific *Drp1* knockout (*Drp1^−/−^*) mice, muscle atrophy and mitochondrial dysfunction were observed [31,32]. Despite an increase in volume, the mitochondria isolated from *Drp1^−/−^* mice showed reduced respiration activity and lower expression levels of complex I and III of the electron transport chain (ETC). The calcium homeostasis of their mitochondria was also disrupted, leading to the upregulation of ubiquitin ligases (e.g., atrogin-1 and MuRF1) that promote muscle atrophy [33]. Hence, a balance of mitofission and mitofusion is essential to optimize the muscle functions.

Degradation of the split mitochondria can be processed in PTEN-induced kinase 1 (PINK1)-dependent or PINK1-independent manner. PINK1 is a mitochondrial membrane protein that undergoes autophosphorylation when the mitochondrial membrane potential is disrupted. Activated PINK1 phosphorylates the E3 ubiquitin ligase Parkin at Ser 65 [34], which subsequently ubiquitinates a number of proteins such as MFNs [35], Tom20 [36], and VDAC [37] on the damaged mitochondria. The ubiquitinated proteins are then recognized by the adaptor protein p62/STQM, which tethers the mitochondria to the microtubule-associated protein 1A/1B-light chain 3 (LC3) on the autophagosome [38,39]. Mitochondrial uptake by autophagosome can also be initiated without the involvement of PINK1. Mitophagy receptors such as BCL2/adenovirus E1B 19 kDa protein-interacting protein 3-like (BNIP3L) or FUN4 domain containing protein 1 (FUNDC1) could be recruited to the depolarized mitochondrial membrane, which forms a linkage directly with lipidated LC3 (LC3-II) on the autophagosomes [38]. Due to the independence of PINK1/Parkin- and mitophagy receptor-mediated mechanisms, they may serve as complementary pathways to maintain mitochondrial quality [40].

**Figure 1:**
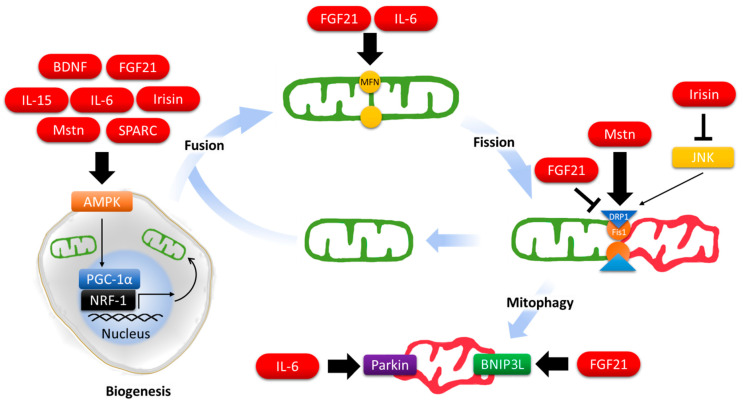
Regulation of mitochondrial dynamics by myokines.

Given the importance of mitochondria in supporting bioenergetics homeostasis, the mitochondrial life cycle is tightly coupled to the cellular concentration of nutrients and ATP via the energy sensors AMPK [41] and mammalian target of rapamycin (mTOR) [42,43]. For instance, AMPK induces mitochondrial synthesis through PGC-1α [44], triggers the formation of autophagosomes by enhancing Unc-51-like kinase 1 (ULK1) activity [45], initiates mitophagy by promoting mitochondrial recruitment of PINK1 [46], and catalyzes mitofission by increasing MFF-DRP1 interaction [47]. Numerous efforts have been put in identifying the signaling cascades and intracellular regulation of the mitochondrial life cycle in the last decades but little is known about how the cells provoke mitochondrial adaptation in response to the extracellular clues. In the following section, we will present examples of myokines that are involved in mitochondrial recycling control (Figure 1).

### 2.1. Brain-Derived Neurotrophic Factor (BDNF)

BDNF is a critical growth factor in neuronal development, neuron survival, and synaptic plasticity [48,49] and is associated with many neurodegenerative diseases including Alzheimer’s disease, Parkinson’s disease, Rett syndrome, etc. [50,51]. BDNF level in circulation and *Bdnf* expression in skeletal muscle were enhanced after physical exercise or food deprivation [52,53,54]. It has been suggested that the skeletal muscle-generated BDNF acted solely as an autocrine because overexpression of BDNF in the muscle did not alter its concentration in blood [53]. Using the muscle-specific BDNF knockout (MBKO) mice, however, we and Fulgenzi et al. have found recently that BDNF can be secreted into circulation [54,55]. We and Matthews et al. have also shown that BDNF is a positive regulator of mitochondrial activity in the cultured muscle cells [53,54]. Hence, BDNF might serve as a myokine that regulates the mitochondrial activity in muscle and other tissues when the animals are metabolically stressed. In support of this notion, we have demonstrated that muscle-generated BDNF is essential to preserve the mitochondrial content in skeletal muscle during fasting [54]. Moreover, chronic activation of BDNF receptor, TrkB, by the non-peptidyl agonist 7,8-dihydroxyflavone (7,8-DHF), increased mitochondrial content in mouse skeletal muscles through activating the AMPK-PGC-1α cascade and alleviated the detrimental effects of diet-induced obesity [56]. BDNF is also able to promote mitochondrial biogenesis in hippocampal neurons [57] and controls mitochondrial dynamics and clearance in adipocytes [58], myocardium [59], motor neurons [60], and retinal cells [61]. Although all these findings suggest that BDNF is a regulator of mitochondrial behavior in a great variety of tissues, it is interesting to note that none of these studies were exercise-related. Hence, the functional significance of BDNF spur during exercise still remains unsolved.

### 2.2. Fibroblast Growth Factor 21 (FGF21)

In *Fgf21*-ablated skeletal muscle, BNIP3L-mediated mitophagy was diminished, suggesting that FGF21 drives mitophagy. This notion was further demonstrated by the augmented mitochondrial breakdown in *Fgf21*-overexpressed cells [62]. Nevertheless, it was found that FGF21 temporarily promotes mitochondrial fusion and inhibits mitofission to prevent apoptosis or senescence when the mesenchymal stem cells (MSCs) are short of energy supply [63]. The role of FGF21 in mitochondrial biogenesis has not been fully explored but there was evidence showing that FGF21 induced mitochondrial replication through PGC-1α and SIRT1 activations [64]. FGF21 also activates the PGC-1α-NRF1-mtTFA pathway to induce mtDNA replication in goat adipocytes [65]. Nonetheless, there is no direct evidence showing that FGF21 increases the mitochondrial number or mitochondrial protein expression in laboratory rodents.

### 2.3. Interleukin 6 (IL-6)

IL-6 poses prominent effects on mitochondrial dynamics in a number of tissues. For instance, it promoted mitochondrial biogenesis in cortical astrocytes [66] and colorectal cancer cells [67]. In myocardium without IL-6 production, compromised mitochondrial dynamics was detected as the cells showed augmented mitochondria size with reduced *Mfn2* expression [68]. Imbalanced mitochondrial production and degradation was found in the skeletal muscle of cancer cachexia, in which IL-6 overexpression in this tissue diminished mitochondrial content but simultaneously elevated the expressions of PGC-1α and Mfns [69]. Fix et al. reported that knocking out the IL-6 receptor reduced mitophagic flux despite augmented mitofission [70], which concurs with the compromised muscle endurance and reduced oxygen consumption in the IL-6 knockout (*IL-6^−/−^*) mice [71]. Because exercise intervention was not able to increase the amount of mitochondrial proteins such as citrate synthase and COX in *IL-6^−/−^* mice [71,72], this myokine might play a role in promoting mitochondrial biogenesis. Indeed, IL-6 stimulated 3T3-L1 adipocytes showed upregulated mitochondria biogenesis as a compensation for the ROS-induced mitochondria loss, suggesting the mitochondrial dynamic regulatory actions of IL-6 is valid in multiple tissues.

### 2.4. Interleukin 15 (IL-15)

Circulating IL-15 level is inversely associated with the overall adiposity in both human [73] and laboratory rodents [74,75]. When the animals received a supraphysiological dose of IL-15 or having IL-15 overexpressed in their skeletal muscle, a lower respiratory exchange ratio and higher whole-body fatty acid oxidation (FAO) were observed [76,77]. Since FAO increased in rat muscle after a short IL-15 incubation, it was suspected that IL-15 mediated the fat-burning function via enhancing the activity of mitochondrial enzymes. Nevertheless, the possibility that mitochondrial biogenesis also contributed to the augmented FAO should not be excluded as IL-15 has a stimulatory effect on the expression of mitochondrial biogenesis-related genes including peroxisome proliferator-activated receptor α (PPARα), PPARδ, PGC-1α, PGC-1β, uncoupling protein 2 (UCP2), and NRF1 in muscle cells [78,79]. The observation of increased mtDNA content after chronic IL-15 stimulation further supported this idea [80]. Interestingly, the high circulating level of IL-15 was still able to increase the mitochondrial content in the muscle of IL-15 receptor knockout mice indicating that IL-15 may mediate its effect via an unknown mechanism other than IL-15 receptor activation [81,82,83].

### 2.5. Irisin

Irisin is an exercise-induced myokine formed by proteolytic cleavage of fibronectin type III domain-containing protein 5 (FNDC5) [84]. It promotes mitochondrial biogenesis in muscle via the AMPK-PGC-1α activation [85], resulting in increased cellular mitochondrial content and oxygen consumption [86]. When *Fndc5* expression was suppressed, on the other hand, the consequential PGC-1α defects lowered the abundance and size of mitochondria [87]. Irisin was also able to retain mitochondrial biogenesis, dynamics, and autophagic program in chondrocytes to repress inflammation-mediated oxidative stress and extracellular matrix underproduction [88]. Interestingly, irisin inhibits mitochondrial fission via the JNK-LATS2 pathway in cardiomyocytes [89] and hepatocytes [90] when excessive fission occurred during inflammation. Presumably, irisin may function as a beneficial effector of exercise to protect against excessive damage induced by inflammation.

### 2.6. Myostatin (Mstn)

Mstn is a known inhibitory myokine to suppress myogenesis [91]. In Mstn knockout (*Mstn^−/−^*) mice, muscle hypertrophy was observed with a higher ratio of glycolytic-to-oxidative myofibers [92,93], suggesting Mstn is also crucial to myofiber type determination. Moreover, the muscle of *Mstn^−/−^* mice contained less mtDNA and displayed smaller mitochondrial volume [94,95]. The underlying mechanism causing these mitochondrial defects has not been identified but there is evidence that Mstn promotes mitochondrial biogenesis through Smad signaling [96]. Interestingly, Mstn also stimulates both mitochondrial breakdown and mitofission via modulating the expression of mediators such as *Drp1* and *Fis1* [97,98], suggesting the mitochondrial recycling mechanism is boosted. However, the physiological implication of this stimulatory function is unknown at the current stage.

### 2.7. Osteonectin/Secreted Protein Acidic and Rich in Cysteine (SPARC)

Similar to IL-15 and irisin, the expression of osteonectin (also known as SPARC) can be induced by exercise [99]. Current studies have only uncovered the activities of osteonectin in undifferentiated myoblasts, where it upregulated mitochondrial proteins such as ubiquinol-cytochrome c reductase core protein II (UQCRC2) and succinate dehydrogenase subunit (SDHB) probably through AMPK activation [100,101]. This osteonectin-dependent elevation of mitochondrial protein is important to myogenesis [100]. Because no study on the role of osteonectin in post-exercise skeletal muscle has been performed, the action mechanism of this myokine in mitochondrial adaptation remains unknown.

## 3. Myokines and Oxidative Stress

ROS are a group of oxygen-derived molecules and free radicals that are constantly generated and scavenged intracellularly. Common ROS include superoxide anion, peroxide, hydroxyl radical, hydroxyl ion, and nitric oxide [102]. Among the organelles, respiring mitochondria are the major ROS production sites. During the conversion of ADP into ATP by the ETC complexes located on the inner mitochondrial membrane, superoxide (O_2_^•−^) is formed at complex I and complex III due to intrinsic electron leakage [103]. The free electrons generated by oxidative phosphorylation may also attack oxygen molecules and thus produce intracellular O_2_^•−^ [104]. These superoxides in the mitochondrial matrix or intermembrane space would be converted into hydrogen peroxide (H_2_O_2_) and ultimately into water molecule (H_2_O) [105]. Both O_2_^•−^ and H_2_O_2_ are considered as mitochondrial ROS (mtROS). ROS can also be formed by cytosolic NADPH oxidase (NOX). Located on the cell membrane, NOX plays a major role in oxidizing NADPH to NADP^+^ in response to signaling stimulation or extracellular stresses. Formation of cellular ROS and mtROS could be a spontaneous response to environmental factors such as ionizing radiation [106,107]. Excessive accumulation of ROS is detrimental to almost all biological macromolecules. The toxic effect of ROS could be demonstrated by their actions in DNA damage, peroxidation of fatty acids and amino acids, and inactivation of enzymes or receptors. For instance, peroxidation of the mitochondrial lipid cardiolipin on the inner mitochondrial membrane causes the release of cytochrome c and reduced ATP production [108]. In addition to organelle proteins, both mtDNA and nuclear DNA are also targets of ROS. Damage of mtDNA is especially devastating as it might lead to mitochondrial protein dysfunction that produces more mtROS, resulting in a vicious cycle of mtROS amplification [109]. Moreover, ROS are prominent inducers of apoptosis via the c-Jun N-terminal kinases (JNK)/p53 pathway [110,111] and necrosis through the formation of receptor-interacting serine/threonine protein kinase (RIP) 1 and 3 complexes [112]. Hence, detoxification of excessive ROS is critical to relieve intracellular oxidative stress and maintain cell survival. Several antioxidant mechanisms are present to scavenge cellular ROS. Superoxide dismutase (SOD) is a multigene family consisting of three isoforms. They convert O_2_^•−^ into H_2_O_2_, which will be further decomposed into water molecules and oxygen by another antioxidant enzyme catalase [113]. Small molecules such as glutathione, a tripeptide composes of cysteine, glycine, and glutamate, also play a crucial role in ROS clearance. Glutathione at reduced state (GSH) turns into the oxidized state (GSSG) when they are attacked by ROS, which could be restored into the reduced state by glutathione reductase (GR) [114]. Hence, the level of GSH represents the ‘antioxidation capacity’ of a cell to protect important cellular structures or molecules from ROS attack.

It is noteworthy that the formation of ROS in trace amounts (i.e., mitohormesis) is necessary as they are essential messenger molecules to convey intra-and inter-cellular signaling. For instance, nitric oxide (^•^NO) is a common extracellular ROS messenger for blood vessel dilation and blood pressure control [115]. Similarly, mtROS is an important second messenger of neurokinin, a neurologically active peptide, in damage-sensing neurons [116]. Intracellular H_2_O_2_ molecules also provide the capacity of converting cysteine residues from thiolate anion (Cys-S^−^) into sulfenic group (Cys-SOH) or even higher oxidative forms, which are important to alter protein conformation and functions [117,118].

Mitochondrial structure and cellular ROS content are bi-directionally linked. On one hand, the stress response signaling pathways induced by ROS activate the mitochondrial fission machinery in a number of tissues including skeletal muscles [119]. On the other hand, the aberrant mechanism in mitochondrial dynamics as observed in the depletion of *Mfn1* or *Mfn2* results in the formation of mtROS [120]. The molecular details that govern or modulate these activities have not been well-established but the post-translational modification of key enzymes (e.g., S-nitrosylation of Drp1) in mitochondrial dynamics might play an indispensable role [121]. Given the interconnection between ROS generation and mitochondrial remodeling, myokines that control mitochondrial dynamics might also serve as the regulators of ROS production in cells. While the regulatory activities of myokines in ROS generation or oxidative stress-defense system have been reported in a number of cell types, surprisingly, their effect on skeletal muscle remains poorly studied. We will discuss the role of myokines that are potential regulators of muscle ROS homeostasis in the following section (Table 1).

### 3.1. BDNF

Numerous studies have demonstrated that BDNF is a protective factor against ROS-induced damage in neurons. Cortical cultures challenged with 3-nitropropionic acid (an irreversible inhibitor of mitochondrial complex II) have high cellular ROS, which could be alleviated by the BDNF-stimulated sestrin2-nitric oxide (NO)-protein kinase G (PKG)-NFκB pathway [122]. Similarly, recombinant BDNF incubation averted the H_2_O_2_-induced cell death in cortical neurons and astrocytes [123], which was believed as an important mechanism to delay neuronal aging [124]. The ROS removal effect of BDNF could also be observed in H_2_O_2_-stressed adult neural stem/progenitor cells (NSPCs) via enhancing the activities of GR and SOD [125]. On the other hand, attenuating the long non-coding RNA (lncRNA)-suppressed BDNF expression lowered the ROS intensity and malondialdehyde content in PC12 cells [126], suggesting BDNF may work as an autocrine to protect the cells from oxidative damage. It has also been reported that hyperglycemia-induced ROS generation in brain microvascular endothelial cells could be inhibited by exogenous BDNF stimulation, which extricated the cells from apoptotic programming [127]. Because ROS elimination prevents neuronal cell apoptosis [125] and boosts survival rate in neural transplantation surgery [128], augmenting BDNF production may represent a compelling method to alleviate the pathological outcomes caused by excessive oxidative stress in the CNS. Recently, BDNF was also found effective in suppressing the oxidized low-density lipoprotein (oxLDL)-induced ROS surge in human umbilical vein endothelial cells (HUVECs) [129]. Hence, it would be tempting to postulate that this function might also be conserved in non-CNS tissues, such as skeletal muscle.

### 3.2. FGF21

FGF21 is a repressor of ROS generation in multiple tissues. HUVECs pre-incubated with recombinant FGF21 were rescued from glucose-induced ROS production and cell death [130]. Another study also reported that FGF21 inhibited the oxLDL-provoked mtROS production in vascular endothelial cells [131], which provides an explanation of how these cells escape from pyroptotic cell death [131,132]. FGF21 treatment in cultured cardiomyocytes enhanced the expression of genes in antioxidative pathways [133]. It has also been reported that FGF21 compromised the hypoxia-mediated ROS synthesis in cerebral microvascular endothelial cells and restored their antioxidants activities [134]. FGF21 is particularly effective in protecting the cells from mitochondria-dependent stress. In MSCs, mtROS accumulation was observed after FGF21 depletion, which resulted in cell senescence at early passage [63]. In addition, FGF21 relieves the oxidative stress of pathological conditions such as alcoholic fatty liver disease that FGF21 pre-incubation or FGF21 injection restored the antioxidant activities of SOD and glutathione peroxidase in ethanol-induced HepG2 cells and alcohol-gavaged mice, respectively [135]. Whether FGF21 also imposes a protective role against oxidative stress in skeletal muscle remains to be determined.

### 3.3. IL-6

In skeletal muscle, IL-6 stimulation increased mtROS production via opening the transition pore in isolated mitochondria and cultured C2C12 myotubes [136]. In IL-6-overexpressed mice, their diaphragm muscles showed increased NOX2 expression, higher ROS accumulation, and diminished NRF2-related antioxidant responses [137]. IL-6 also acts as an oxidative stress inducer in various cell types, particularly in cancer cells. It promotes cell proliferation by activating the mitochondrial single strand DNA binding protein (mtSSB) in colorectal cancer cells, resulting in accelerated mitochondrial biogenesis and mtROS over-production [67]. In hepatocellular carcinoma cells, IL-6 treatment generated intracellular ROS via the pathways of JNK, disintegrin and metalloproteinase domain-containing protein 9, and NOX1, which is important to epithelial-mesenchymal transition [138,139]. Moreover, several reports have suggested that IL-6 increased ROS levels in 3T3-L1 adipocytes [140], TIG3 fibroblasts [141], and human brain microvascular endothelial cells [142]. These findings demonstrate that IL-6 is a pivotal ROS up-regulator in numerous conditions and cells. However, there is also evidence showing that IL-6 lowered the cellular ROS by promoting removing the antioxidant repressor KEAP1 in pancreatic β-cells [143], suggesting that IL-6 might have opposite functions according to the cell type.

### 3.4. IL-15

IL-15 is a ROS scavenger that protects immune cells from immunological response-derived oxidative stress. In human T cells, ROS-induced apoptosis is prevented by the upregulation of GR, thioredoxin (TXN) reductase 1, peroxiredoxin, and SOD after IL-15 stimulation [144]. A recent study also reported that IL15-primed natural killer (NK) cells are shielded from oxidative stress, in which IL-15 boosts the TXN expression and reducing mitochondrial localization of TXN-interacting protein via mTOR activation. These IL-15-primed NK cells also present denser cell surface thiol groups for counteracting the ROS cytotoxicity [145]. In cultured muscle cells, H_2_O_2_-induced oxidative stress could be relieved by IL-15 pre-incubation [146]. Because no other study regarding the role of IL-15 in muscular oxidative stress regulation is available, it remains unknown if IL-15 modulates the ROS level of mouse muscle in vivo.

### 3.5. Irisin

Although adenovirus-mediated overexpression of irisin in mouse cardiomyocytes increased cellular level of superoxide [147], it alleviated the oxidative stress in the heart and protected the tissue from ischemia/reperfusion injury. This protective effect relies on SOD activity promotion, and restoration of SOD into the mitochondrial compartment [148]. It has also been reported that irisin lowered the cellular ROS content by upregulating mitochondrial uncoupling protein 2 (UCP2) in both ischemia/reperfusion-injured alveolar cells [149] and hepatocytes [90]. The oxLDL-promoted ROS generation in vascular endothelial cells could be reversed by irisin through Akt/mTOR/NRF2 pathway activation [150]. Surprisingly, irisin increased the ROS generation activating AMPK-induced glucose uptake in L6 muscle cells [151]. It remains unknown if this ROS generation is a part of mitohormesis in muscle and more studies are needed to reveal the role of this important myokine in skeletal muscle metabolism.

### 3.6. Mstn

There are a number of studies showing that Mstn is a ROS-inducing myokine in skeletal muscles. Mstn treatment resulted in a drastic increase of intracellular ROS content of C2C12 myotubes [152]. This ROS generation is initiated via the MAPKs (p38 and ERK) signaling pathway, which ultimately triggered oxidative stress-dependent muscle wasting [153]. Activities of the antioxidants catalases were significantly reduced in both gastrocnemius and soleus of *Mstn^−/−^* mice but enhanced GSH amount, higher glutathione peroxidase activity, and decreased glutaredoxin activity were observed only in their gastrocnemius, suggesting Mstn suppresses muscular antioxidant systems in a myofiber-specific manner [94]. Mstn also regulates intracellular oxidative stress in non-muscle tissues. For instance, ablation of *MSTN* in HeLa cells promoted fatty acid oxidation-related mtROS generation and caused mitochondria-dependent apoptosis [154]. Alternatively, recombinant Mstn treatment induced a ROS level surge alongside the upregulated *NOX* expression in proximal tubular epithelial cell [155]. These findings indicate that Mstn is an inducer of intracellular oxidative stress in various tissue types.

## 4. Therapeutic Potential of Myokines in Mitochondria/Ros ROS Dysregulation

Defective mitochondrial dynamics and ROS imbalance are observed in pathological conditions including metabolic disorders, immunological disease, neurodegenerative disease, cancer development, and aging [156,157,158,159,160,161,162]. Thus, the use of chemical agents that promote new mitochondrial synthesis or removing the damaged mitochondria should be beneficial to the health of these patients. Proof-of-principle evidence is available that accelerating the mitochondrial dynamics by chemical agents such as AMPK agonist 5-aminoimidazole-4-carboxamide ribonucleotide (AICAR) effectively alleviated the pathological symptoms of disease with mitochondrial dysfunction [163]. Similarly, strengthening the defense system that opposes excess ROS production during disease progression is a hit target of research. For instance, the administration of an antioxidant peptide SS31 promoted mtROS elimination during cardiac IR injury [164,165]. Because of their regulatory roles in mitochondrial activity regulation, the application of myokines should have a great potential to treat mitochondrial-related diseases, in particular since most myokines have promising effects in multiple tissues. Because numerous excellent reviews have already discussed the muscle-tissues crosstalk via myokine in health maintenance [166,167,168,169,170], we will here present evidence to show the potential use of myokines as an “exercise pill” and an anti-aging drug.

### 4.1. Can Myokines Be Used as an Exercise Mimetic?

Exercise has been recognized by the World Health Organization (WHO) as an effective intervention to prevent or alleviate various chronic diseases [171]. Endurance exercise training also provides cytoprotection to skeletal muscle fibers against damaging insults such as chemical-induced injury and inactivity-induced atrophy [172]. Although the mechanism of exercise-provoked benefits has not been fully elucidated, it is hypothesized that mitochondrial remodeling plays a central role because exercise promotes mitochondrial turnover in skeletal muscles [173]. Extensive studies have shown that exercise training, regardless of the intensity and duration, increases the mitochondrial mass [174], mtDNA content [175], FAO [176,177], and mitochondrial volume density in skeletal muscle [178]. Post-exercise upregulation of mitophagy is also required to ensure sufficient clearance of the damaged mitochondria, which depends on the AMPK-ULK1 signaling [179]. Because myokine expression in skeletal muscle is increased after exercise [180,181], and that myokines participate in multiple regulatory points of mitochondrial homeostasis, they are thus naturally linked to the metabolic and functional benefits of exercise. A canonical example stated that SPARC is the essential effector of exercise to suppress colon tumorigenesis [99]. The finding that irisin injection was able to prevent bone loss and muscle atrophy in hind-limb suspended mice further encourages the use of myokines as an exercise mimetic to provide health benefits to those subjects with locomotion impairments [182,183]. However, it should be noted that hundreds of myokines are induced during exercise and it is hard to be convinced that increasing a single myokine in vivo would significantly mimic all beneficial effects of exercise. Infusion of exosomes, the nano-vesicles that contain a mixture of multiple myokines, miRNA, and nucleic acids derived from young exercised muscle [184], may represent an alternative to single myokine administration, which in principle has a higher translational value. Nevertheless, the variation of exercise intensity and duration might produce a highly heterogeneous set of exosomes with unmanageable effects, and that their action mechanisms in various tissues have not been well characterized. More research on exosome biology is thus needed before it becomes a choice of therapeutic interventions.

It is also interesting to foresee that myokines can be used to boost exercise performance because genetic ablation studies have demonstrated the essential role of some myokines in muscle endurance [54]. Increased mitochondrial biogenesis is believed to play a major role in enhancing the energetic capacity of skeletal muscle [185,186]. As such, it is not surprising to find that the overexpression of signaling molecules such as PGC-1α or myokines like musclin improves the exercise performance of mice [187]. Those myokines such as IL-6 and IL-15 that are able to enhance FAO in skeletal muscle might also have performance enhancing functions such as shifting the energy source from carbohydrates to fatty acid during exercise, which is essential to extend the muscular endurance capacity [188,189].

Because of their antioxidant activity, some myokines could also be applied to protect against muscle fatigue. It is well-known that muscle contraction promotes the synthesis of ROS and free radicals [190,191], which are associated with muscle fatigue [192]. The application of anti-oxidants that protect against mtROS accumulation in muscle is able to delay muscle exhaustion, indicating that ROS scavengers are useful in relieving tissue tiredness [193]. Nevertheless, the regular generation of ROS at a moderate level is essential to redox homeostasis for enhancing the capacity of the antioxidant defense system in the exercised muscle [194]. A recent study also proposed that myocellular ROS production contributed to GLUT4 translocation [195], which is a key modulator of exercised-stimulated glucose uptake in muscle [196]. As such, myokine administration should not be used as a daily supplement but as a recovery measure after strenuous exercise or over-training when the detrimental effects of ROS are exaggerated [197].

### 4.2. Can Myokines Be Used as Rejuvenation Agents?

Mega-analysis suggests that as much as 83% of the elderly are physically inactive [198]. Such immobility can be explained by the deterioration of muscles including muscle mass decreases, reduced muscle strength, and loss of functional capacity [199,200]. If the muscle is not stimulated by sufficient levels of physical activity, the synthesis and release of myokines will be limited, which may contribute to the malfunction of several organs. Indeed, physical idleness is associated with reduced myokine secretion. For instance, the contents of bone morphogenetic factor 7 (BMP7) and irisin are decreased in aged skeletal muscle, which could be restored by physical exercise [201,202]. Because BMP7 is important to counteract the aging-dependent dysfunction of neuromuscular junctions [203], its deficiency may contribute to the sarcopenic phenotypes of the elderly. It is also found that progenitor/satellite cells isolated from aged rat skeletal muscles underwent increased internalization of SPARC, resulting in less muscle-generated SPARC. Such a decline in SPARC level caused muscle adipogenesis and fat accumulation [204]. Other myokines including IL-15, β-aminoisobutyric acid (BAIBA), and IGF-1 were also decreased in aged subjects [205]. Because myokines such as irisin, FGF21, and decorin are important in maintaining muscle mass [206], the lack of sufficient myokines in the elderly may form a looping back model to magnify muscle atrophy and dynapenia.

In a gerontological study that compared the vastus lateralis biopsies collected from young (average age = 22 years) and old (average age = 72 years) males, it was found that oxidative damage was more severe in the aged group [207]. A similar rise of H_2_O_2_ and O_2_^•−^ were also detected in old rat muscles [208], suggesting the increases of muscular oxidative stress during aging is a universal event among different species. Moreover, mitochondrial activity is lower in the skeletal muscle of aged mice [209]. Their muscles are thus less potent in eliminating the impaired ROS-generating mitochondria, which leads to more ROS generation [210,211]. The cause of this elevated ROS production has not been clearly elucidated because discrepant observations were reported. While a drop of catalase protein level has been proposed as the major contributor of the elevated oxidative stress in the gastrocnemius of middle-aged to old mice [212], there are also studies reporting that muscle catalase activity was increased in an age-dependent manner [207,213,214]. Similarly, SOD content upon aging is inconsistent with reports that the muscles from healthy aged human subjects displayed higher SOD activity [207,215]. A decrease of SOD2 activity in sedentary old adults [216] and in 12- or 24-months old mice [212] has also been reported.

Although myokine levels have a close-relationship with aging, its anti-aging effect has not been systemically evaluated. Fragmented but yet encouraging results were reported that intramuscular injection of decorin significantly induced muscle hypertrophy in pre-clinical models of muscle dystrophy [217]. Reza et al. have also shown that irisin was able to promote myogenesis and muscle regeneration [218], which are both severely impaired in the elderly [219,220]. Moreover, administration of myostatin-neutralizing antibody increased muscle mass and muscle strength in aged mice [221]. Because myokines such as myostatin and IGF-I were independently associated with muscle wasting [222], the use of myokine as a biomarker of frailty, a reversible state of increased vulnerability to health-related negative outcomes, including disability and mortality, was proposed [223]. Indeed, the close association of irisin or BDNF levels with the sarcopenia index suggested that these myokines can be used as a reliable biomarker of sarcopenia and frailty [224,225].

## 5. Conclusions and Future Perspectives

The identification of myokines has provided a new conceptual basis and paradigm on the regulation of metabolism but our knowledge on this new hormone class is still incomplete. Because deficiency of some myokines is highly associated with diseases such as Alzheimer’s disease [226,227] or metabolic syndromes [228], the large number of potential myokines in muscle provides a valuable resource for disease prognosis and treatment. They can also be used as biomarkers for assessing the type and amount of exercise that are required for the prescription of exercise for people with chronic diseases [229]. Nevertheless, most myokines are still uncharacterized, which limits their translational potential. An immediate challenge in the field is to accelerate the functional studies of these factors systematically. The use of reverse genetic methods is essential, which has greatly advanced our understanding of myokines’ functions in the last decades. However, the knockout models of rarely-studied or novel myokines are not always available. Collaboration with the International Mouse Phenotyping Consortium (IMPC), a central platform that aims to knockout each of the roughly 20,000 genes of the mouse genome, might provide great support to facilitate the myokine characterizations [230].

The use of exogenous hormone/peptide supplementation is another well-accepted method to delineate the function of myokines. Most studies performed in laboratory animals require injections of myokines in high dose, which is a less desirable method for therapeutic purposes in human because of the peptidyl limitations such as proteolytic instability, low solubility, short half-life in plasma, and toxicity in a high dose of myokines. Ideally, non-peptidyl bioavailable mimetics should be used but there is only a limited number of myokine mimetics have been developed. To our best knowledge, only the non-peptidyl mimetics for FGF21 and BDNF have been proved as beneficial agents to metabolic syndromes and neurological disorders [56,231]. More resources should be invested to develop reliable and potent mimetics for those myokines with translational potential in the future.

Although a lot of peptides are classified as myokines, most of them are commonly expressed in various tissues rather than generated solely by skeletal muscle. For instance, IL-6 expression could be detected in macrophages [232], cancer cells [232], osteoblasts [233], smooth muscle cells [234], and adipocytes [235]. This leads to the concern on the source of myokines secreted in response to stress because the tissue-specific function of myokines might jeopardize their potential application in health improvement. IL-6 is one of the classic examples that the IL-6 produced in muscle enhances glucose disposal [236], lipolysis, and fat oxidation [188] but the adipocyte-derived IL-6 promoted insulin resistance [235]. Presumably, simply elevating the circulating myokine levels may not be beneficial if it possesses contradictory functions in different tissues. A full revelation of the functional spectrum of myokines that are also expressed in other tissues is a must before they can be considered for clinical usage.

## Figures and Tables

**Table 1:** Oxidative stress regulation by myokines in different cells/tissues.

Myokine	Cell/Tissue Type	Treatment/Pathological Condition	Oxidative Stress Regulation	Reference
BDNF	Cortical neurons	3-Nitropropionic acid challenge	Alleviates ROS level	[122]
	NSPCs	H_2_O_2_ stress	Enhances the activities of GR and SOD	[125]
	PC12	/	Lowers ROS intensity and malondialdehyde content	[126]
	Brain microvascular endothelial cells	Hyperglycemia	Inhibits ROS generation and prevents apoptosis	[127]
	HUVECs	oxLDL incubation	Suppresses ROS surge	[129]
FGF21	HUVECs	High glucose concentration incubation	ROS elimination	[130]
	Vascular endothelial cells	oxLDL incubation	Inhibits mtROS production	[131,132]
	Cardiomyocytes	/	Upregulates antioxidative genes	[133]
	Cerebral microvascular endothelial cells	Hypoxia	Inhibits ROS synthesis and restores antioxidants’ activities	[134]
	MSCs	/	Reduces mtROS level	[63]
	HepG2 cells/liver	Ethanol incubation/alcoholic fatty liver disease	Restores activities of SOD and glutathione peroxidase	[135]
IL-6	C2C12 myotubes	/	Increases mtROS production	[136]
	Diaphragm muscles	IL-6 overexpression	Increases NOX2 expression, promotes ROS accumulation, and diminishes NRF2-related antioxidant responses	[137]
	Colorectal cancer cells	Cancer	Promotes mtROS synthesis	[67]
	Hepatocellular carcinoma cells	Cancer	Increases ROS generation	[138,139]
	3T3-L1 adipocytes	/	Enhances ROS level	[140]
	TIG3 fibroblasts	/	Enhances ROS level	[141]
	Human brain microvascular endothelial cells	/	Enhances ROS level	[142]
	β-cells	/	Lowers ROS level, removes the antioxidant repressor KEAP1	[143]
IL-15	Human T cells	/	Upregulates antioxidative genes	[144]
	NK cells	/	Preserves TXN and increases thiol group density on cell surface	[145]
	Muscle cells	H_2_O_2_ incubation	Relieves oxidative stress	[146]
Irisin	Cardiomyocytes	Irisin overexpression and ischemia/reperfusion injury	Increases superoxide level, promotes SOD activity and localization in mitochondria	[147,148]
	Alveolar cells and hepatocytes	Ischemia/reperfusion injury	Lowers ROS content	[90,149]
	Vascular endothelial cells	oxLDL incubation	Reduces ROS generation	[150]
	L6 muscle cells	/	Induces ROS generation	[151]
Mstn	C2C12 myotubes	/	Increases ROS content	[152,153]
		*Mstn* knockout mice		[94]
	Gastrocnemius and soleus muscles		Maintains the activities of several antioxidants	
		*MSTN* knockout		[154]
	HeLa cells		Promotes mtROS generation	
	Proximal tubular epithelial cell	/	Raises ROS level and upregulates *NOX* expression	[155]

Abbreviations: GR: glutathione reductase; HUVEC: human umbilical vein endothelial cells; MSCs: mesenchymal stem cells; NK: natural killer; NOX2: NADPH oxidase 2; NSPC: neural stem/progenitor cells; oxLDL: oxidized low-density lipoprotein; SOD: superoxide dismutase; TXN: thioredoxin.
